# NEDD9 Stabilizes Focal Adhesions, Increases Binding to the Extra-Cellular Matrix and Differentially Effects 2D versus 3D Cell Migration

**DOI:** 10.1371/journal.pone.0035058

**Published:** 2012-04-11

**Authors:** Jessie Zhong, Jaime B. Baquiran, Navid Bonakdar, Justin Lees, Yu Wooi Ching, Elena Pugacheva, Ben Fabry, Geraldine M. O'Neill

**Affiliations:** 1 Children's Cancer Research Unit, Kids Research Institute, The Children's Hospital at Westmead, Westmead, New South Wales, Australia; 2 Discipline of Paediatrics and Child Health, The University of Sydney, Sydney, New South Wales, Australia; 3 Department of Physics, University of Erlangen-Nuremberg, Erlangen, Germany; 4 Mary Babb Randolph Cancer Center (MBRCC), West Virginia University, Morgantown, West Virginia, United States of America; Dresden University of Technology, Germany

## Abstract

The speed of cell migration on 2-dimensional (2D) surfaces is determined by the rate of assembly and disassembly of clustered integrin receptors known as focal adhesions. Different modes of cell migration that have been described in 3D environments are distinguished by their dependence on integrin-mediated interactions with the extra-cellular matrix. In particular, the mesenchymal invasion mode is the most dependent on focal adhesion dynamics. The focal adhesion protein NEDD9 is a key signalling intermediary in mesenchymal cell migration, however whether NEDD9 plays a role in regulating focal adhesion dynamics has not previously been reported. As NEDD9 effects on 2D migration speed appear to depend on the cell type examined, in the present study we have used mouse embryo fibroblasts (MEFs) from mice in which the NEDD9 gene has been depleted (NEDD9 −/− MEFs). This allows comparison with effects of other focal adhesion proteins that have previously been demonstrated using MEFs. We show that focal adhesion disassembly rates are increased in the absence of NEDD9 expression and this is correlated with increased paxillin phosphorylation at focal adhesions. NEDD9−/− MEFs have increased rates of migration on 2D surfaces, but conversely, migration of these cells is significantly reduced in 3D collagen gels. Importantly we show that myosin light chain kinase is activated in 3D in the absence of NEDD9 and is conversely inhibited in 2D cultures. Measurement of adhesion strength reveals that NEDD9−/− MEFs have decreased adhesion to fibronectin, despite upregulated α5β1 fibronectin receptor expression. We find that β1 integrin activation is significantly suppressed in the NEDD9−/−, suggesting that in the absence of NEDD9 there is decreased integrin receptor activation. Collectively our data suggest that NEDD9 may promote 3D cell migration by slowing focal adhesion disassembly, promoting integrin receptor activation and increasing adhesion force to the ECM.

## Introduction

Cell adhesion is a prime determinant of the rate of cell migration speed on 2D surfaces [1]. This is a biphasic effect with both too much and too little adhesion resulting in decreased migration speed and persistence. In addition to the contribution that the concentration of extra-cellular matrix plays in cell speed, a role for focal adhesion dynamics has also emerged. At the focal adhesions, clusters of integrin receptors are bound on the external surface to extra-cellular matrix ligands and are associated with filaments of polymerized actin in the cytoplasm. The resulting tensile force generated at the focal adhesions in turn governs integrin activation and cell motility. Thus, the rate of focal adhesion assembly and disassembly contributes to 2D cell migration speed [2]–[6].

Investigations of cell migration have revealed a number of different migration strategies that are available to cells [7]. The mesenchymal mode is characterized by dependence on the dynamic formation and disassembly of focal adhesions [8]. Recent studies have revealed that NEDD9 is a major signalling intermediary in mesenchymal mode migration [9] and although it has long been know that NEDD9 localizes to focal adhesions [10], [11], the role that NEDD9 plays in regulating focal adhesion dynamics has not previously been reported. NEDD9/HEF1/Cas-L is a member of the Cas family of proteins, grouped together based on a conserved overall protein-protein interaction domain structure [12]. Other members of the family include p130Cas/BCAR1, Efs/Sin and HEPL. The Cas proteins are adhesion docking proteins and through extensive interaction with signalling partners these proteins play a role in a variety of biological process, including cell migration. In particular, elevated NEDD9 expression is detected in a range of metastatic cancers and NEDD9 is considered to be a key pro-metastatic protein [13].

Mouse embryo fibroblasts (MEFs) derived from genetically depleted mouse models of the focal adhesion molecules paxillin, FAK and p130Cas were used to establish that each of these molecules stimulates focal adhesion disassembly [6], [14], [15] and these cells exhibit reduced speed [16]–[18]. By contrast, vinculin stabilizes focal adhesions [19], [20] and fibroblasts lacking vinculin expression have more rapid locomotion in 2D migration assays [21]. We have previously shown that short peptides of NEDD9 dominantly interfere with focal adhesions, causing adhesion disassembly and cell rounding [11] and more recently NEDD9 depletion has been associated with increased 2D migration in breast epithelial cells [22] and granulocytes [23]. In contrast, in other cell backgrounds NEDD9 depletion inhibits cell migration [24]–[29]. Thus it appears that NEDD9 may differentially regulate 2D migration speed and focal adhesion dynamics depending on cell type. Therefore, it is important to establish the role of NEDD9 in focal adhesion dynamics in MEFs, to allow comparison with reported effects of other focal adhesion proteins, without the potentially confounding influence of cell-type specific effects.

In contrast to the well-developed models of migration mechanisms in 2D, the understanding of cell migration in 3D and the role of focal adhesion molecules in this process is comparatively less well understood. Indeed, while fibroblasts with genetically deleted vinculin have increased 2D cell speed, conversely these cells have decreased speed in 3D collagen gels [21] and other adhesion proteins similarly give different 2D versus 3D migration outcomes [30]. In 3D environments migrating cells must overcome a different set of physical constraints than those in a 2D environment. Notably, not only does vinculin appear to stabilize focal adhesions, it also regulates cell adhesion force to the ECM and increases traction forces to the ECM [21]. Together, these functions of vinculin are likely to regulate the ability of the cell to negotiate the physical barriers of the 3D environment. The role of NEDD9 as a key signalling intermediary in mesenchymal cell invasion that relies on adhesion to the ECM suggests the possibility that NEDD9 may also regulate adhesion force to the ECM.

In the present study we employ NEDD9−/− MEFs to show that NEDD9 stabilizes focal adhesions and increases β1 integrin receptor activation and adhesion force to the ECM. We show that NEDD9 depletion from fibroblasts increases 2D migration speed and conversely, significantly decreases 3D speed and migration persistence. Together our data reveal an important new role for NEDD9 in the stabilization of adhesions and adhesion force to the ECM.

## Results

### NEDD9 suppresses focal adhesion disassembly

**Figure 1:**
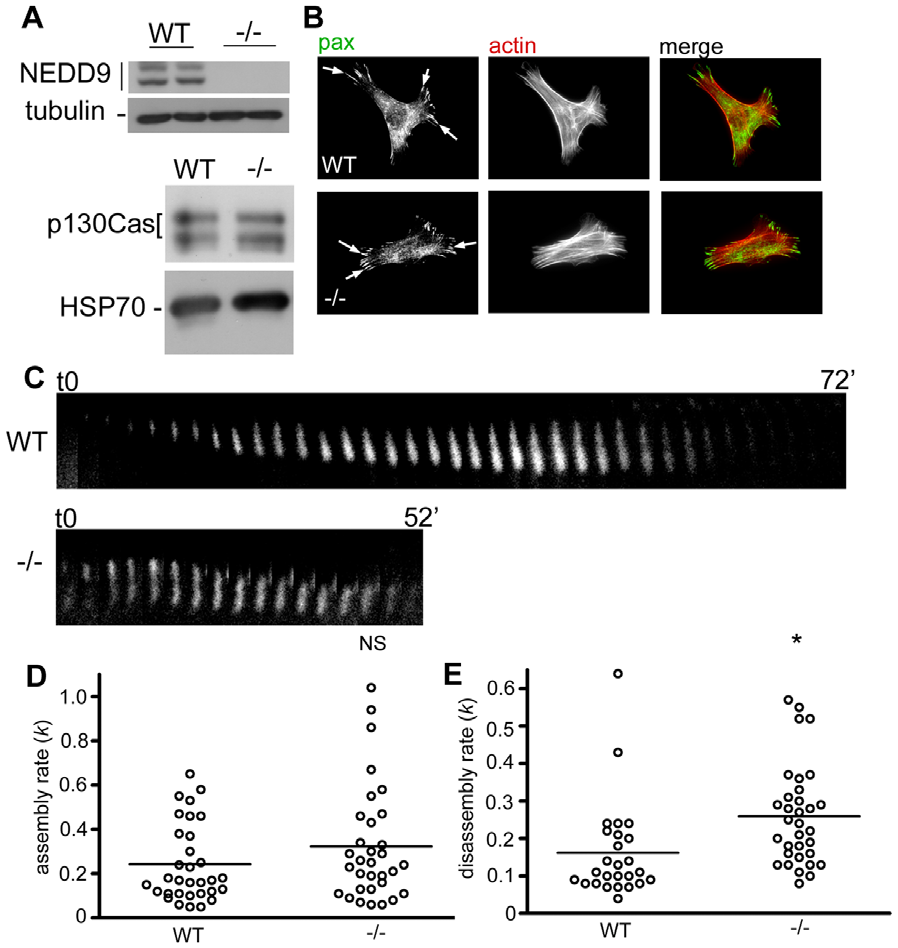
Focal adhesion turnover is more rapid in the absence of NEDD9 expression. A. Western blot analysis of extracts of wild-type (WT) and NEDD9 knockout (−/−) MEFs. Blots were probed with antibodies to NEDD9 which detects the 105 kD and 115 kD phospho-forms of NEDD9 and p130Cas antibodies as indicated. Blots were then probed with anti-tubulin or anti-HSP70 antibodies to confirm equal loading. B. WT and NEDD9 −/− MEFs immunostained with antibodies to paxillin (pax) to detect focal adhesions (white arrows) and TRITC-phalloidin to detect actin stress fibres. C. Examples of YFP-paxillin positive focal adhesions in transfected WT (72 minute lifetime) and NEDD9−/− MEFs (52 minute lifetime) are shown. D. Focal adhesion assembly rate constants (*k*) for cells transfected with YFP-paxillin (n = 32 focal adhesions per cell line). Horizontal bars show the average value per cell line. E. As for D, except data showing the disassembly rate constants (*k*). (WT n = 26, −/− n = 33). *p<0.01, N.S. = not significant.

To address whether NEDD9 plays an active role in regulating focal adhesion turnover we used mouse embryo fibroblasts (MEFs) expressing endogenous wild-type NEDD9 (WT) and NEDD9−/− MEFs (Figure 1A). Due to the high degree of similarity between NEDD9 and p130Cas and the known role of p130Cas in stimulating focal adhesion disassembly in MEFs, we checked for changes in p130Cas expression the NEDD9−/− MEFs. Importantly, there was no difference in either the expression level or the phospho-forms of p130Cas (Figure 1A), thus suggesting there is no compensatory changes in p130Cas in NEDD9−/− MEFs. Immunofluorescence analysis of fixed cells revealed robust focal adhesion and stress fibre formation in both WT and NEDD9 −/− MEFs (Figure 1B), indicating that NEDD9 is not essential for the formation of focal adhesions and stress fibres. We next directly compared the rates of focal adhesion formation and disassembly in cells transfected with YFP-tagged paxillin. Rapid focal adhesion disassembly was observed in regions of protruding membrane in NEDD9 −/− MEFs. By contrast in WT MEFs adhesion disassembly at leading edge appeared slower, with a period of focal adhesion stability preceding membrane extension. Quantification of the assembly and disassembly rates revealed that while assembly rates were essentially equivalent between the two cell lines (Figure 1C and 1D), disassembly rates were significantly faster in the NEDD9 −/− MEFs (Figure 1C and 1E, *p*<0.001). We confirmed that there was no difference in the peak YFP-paxillin fluorescence intensity at focal adhesions in the WT versus NEDD9 −/− MEFs (Figure S1) thus differences in adhesion disassembly rates were not due to differences in exogenous YFP-paxillin expression between the cell lines.

**Figure 2:**
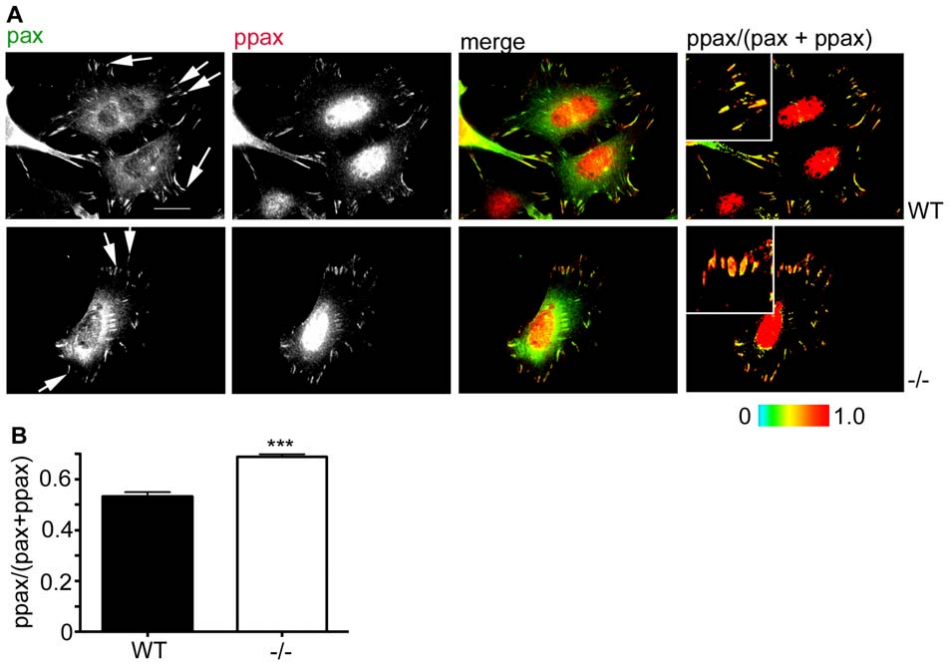
Paxillin phosphorylation is increased in NEDD9 −/− MEFs. A. Paxillin (pax) and phospho-paxillin (ppax) immunostaining. Merged images show colour overlays of paxillin (green) and phospho-paxillin (red). Right hand panels show ratio images of paxillin phosphorylation. Red hues reflect regions of highest phosphorylated paxillin. Boxed insets shows magnified focal adhesions. B. Ratio of phosphorylated paxillin at focal adhesions in WT (n = 197 from 10 individual cells) and NEDD9 −/− MEFs (n = 201 from 10 individual cells). ***p<0.0001, Students' *t*-test.

Based on the increased rate of paxillin-positive adhesion disassembly in the NEDD9−/− MEFs we questioned whether paxillin phosphorylation was increased in NEDD9−/− MEFs, as this is associated with increased focal adhesion turnover [4], [14]. Fluorescence ratio imaging of fixed WT and −/− MEFs co-immunostained with paxillin and phospho-paxillin antibodies revealed increased paxillin phosphorylation at focal adhesions in NEDD9−/− MEFs (Figure 2A). Calculation of the ratio of phoshorylated paxillin at individual adhesions confirmed significantly increased paxillin phosphorylation in the NEDD9−/− MEFs (Figure 2B, p<0.0001). These data are therefore consistent with the idea that the paxillin focal adhesion disassembly switch is more active in NEDD9−/− MEFs.

### NEDD9 differentially regulates 2D versus 3D cell migration

**Figure 3:**
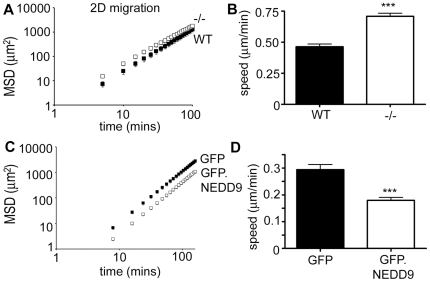
NEDD9 −/− MEFs have increased migration speed in 2D cell migration assays. A. MSD calculated from trajectories of WT (black squares), and NEDD9−/− (white squares) MEFs in 2D cell migration assays. Data are the average values from 3 independent experiments, n>45 cells tracked per experiment. B. Cell speed is significantly increased in NEDD9−/− MEFs (WT n = 150, −/− n = 143). C. MSD calculated from the trajectories of GFP-transfected NEDD9−/− MEFs (black squares) and GFP.NEDD9 transfected NEDD9−/− MEFs (white squares). D. GFP.NEDD9 expression significantly reduces the migration speed of NEDD9−/− MEFs. (n = 53 each). *** *p*<0.0001, Students' *t*-test.

Increased rates of focal adhesion disassembly and paxillin phosphorylation in NEDD9−/− MEFs together suggested that these cells might migrate more rapidly in 2D cell migration assays. Indeed, tracking of individual cells and quantification of the Mean Square Displacement (MSD) revealed that the NEDD9−/− MEFs have significantly faster 2D migration speed (Figure 3A and 3B). To confirm that these effects are directly due to the absence of NEDD9 we performed a rescue experiment with exogenously expressed GFP-tagged NEDD9. Importantly, exogenous GFP.NEDD9 significantly reduced 2D cell migration speed when compared with GFP control transfected NEDD9−/− MEFs (Figure 3C and 3D).

**Figure 4:**
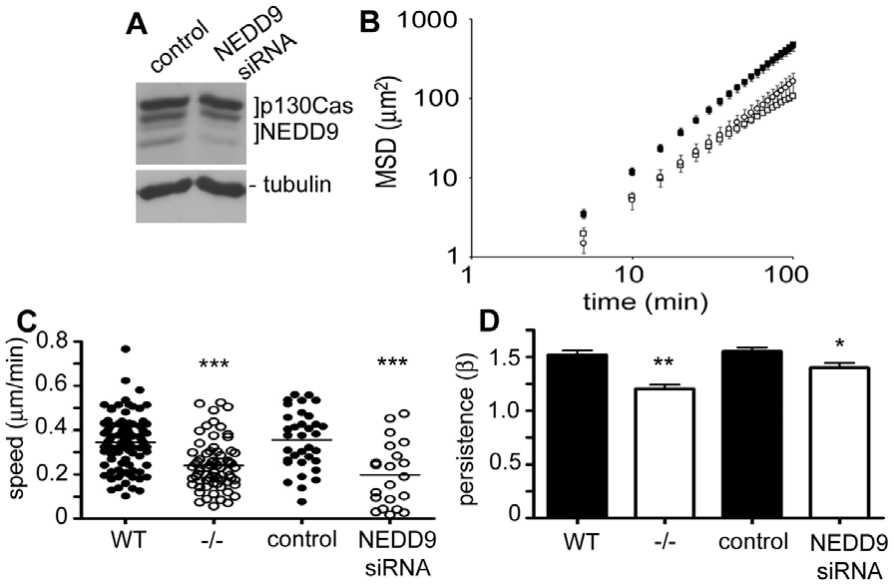
NEDD9 increases 3D migration speed. A. Western blot analysis of cell extracts following treatment with siRNA targeting NEDD9 or control siRNA sequences. Blots were probed with antibodies to NEDD9 which detects 105 kD and 115 kD NEDD9 phospho-forms and cross-reacted with p130Cas as indicated. Probing with anti-tubulin antibodies confirmed equal loading. B. MSD calculated from trajectories of WT MEFs (black squares, average of 3 independent experiments), NEDD9−/− MEFs (white squares, average of 3 independent experiments n>22 cells per experiment), MEFs treated with control siRNA (black circles, average of n = 38 cells) and NEDD9 siRNA (white circles, average of n = 25 cells). Note that identical values were obtained for the WT MEFs and WT MEFs treated with control siRNA; these data points are therefore super-imposed on the graph. C. Cell speed is significantly reduced in NEDD9−/− MEFs and in NEDD9 siRNA treated cells relative to the matched controls. Horizontal bars indicate the average speed for all cells tracked. D. Cell persistence is significantly reduced in NEDD9−/− and NEDD9 siRNA treated MEFs. Histogram shows the average persistence for all cells tracked. *p<0.05, ** *p*<0.001, *** *p*<0.0001, Students' *t*-test.

We next determined the role of NEDD9 in the 3D migration of MEFs, comparing NEDD9 WT MEFs, NEDD9−/− MEFs, and MEFs either exposed to scrambled siRNA controls (control) or following treatment with siRNA targeting NEDD9 expression (NEDD9 siRNA). Control western blot analysis confirmed depletion of NEDD9 protein following NEDD9 siRNA treatment and no effect on p130Cas (Figure 4A). Analysis of the MSD demonstrated that both genetic deletion of NEDD9 (−/−) and NEDD9 depletion with siRNA (NEDD9 siRNA) significantly reduced the migration of MEFs in 3D collagen gels (Figure 4B). The wild-type MEFs and control siRNA-treated MEFs migrated through the 3D collagen gels with greater cell speed (p<0.0001, Figure 4C) and tended to have increased migration persistence (Figure 4D). Thus, in contrast to the effects seen in 2D, we find that 3D cultures cells lacking NEDD9 expression have reduced cell migration speed.

**Figure 5:**
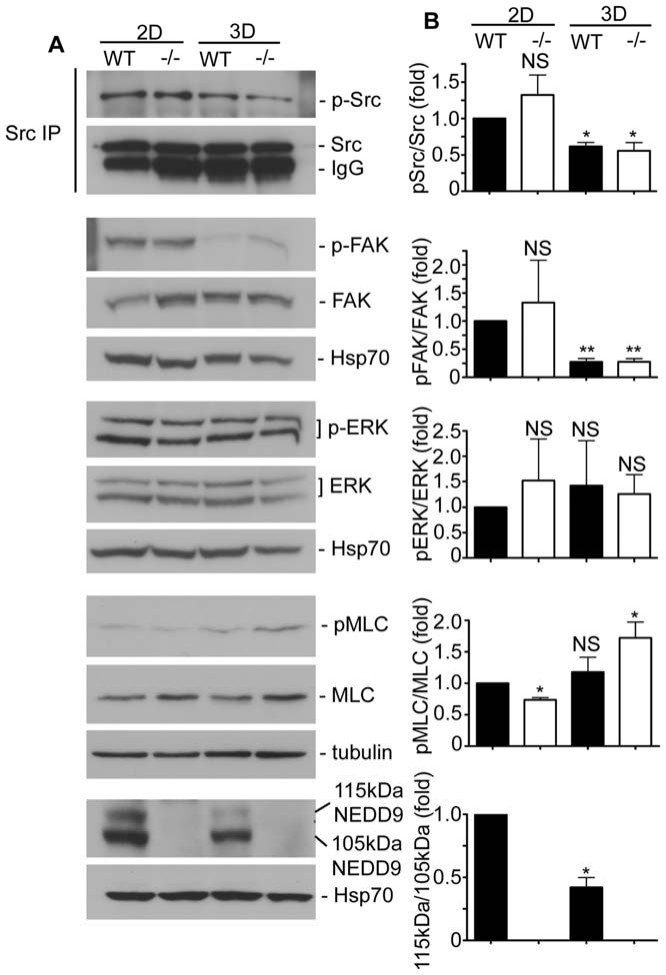
Adhesion signalling pathways are differentially activated in 2D versus 3D. A. Western blot analysis of lysates extracted from WT MEF and NEDD9−/− MEF grown on tissue culture plastic dishes (2D) or in collagen gels (3D). Blots were probed with the indicated antibodies to total proteins and phosphorylated proteins (-p-). Note that total Src was immunoprecipitated (IP) prior to probing with anti-p-Src antibodies. Blots were probed with Hsp70 or tubulin as a loading control. B. Histograms showing densitometric measurements of the level of phosphorylated protein divided by the matching total protein amount and expressed relative to the level in WT MEFs under 2D culture conditions. Data represent the average of triplicate repeats on separate days and error bars show the S.E.M. *p<0.05, ** *p*<0.001, NS = not significant, Students' *t*-test.

Previous reports have demonstrated that Src, FAK, ERK and MLCK are important regulators of focal adhesion dynamics [15]. Due to the striking difference in motility effects in 2D versus 3D culture conditions, we therefore employed phospho-antibodies to analyse the activity of these kinases. This revealed distinct profiles of kinase activity between cells cultured in 2D and 3D. While ERK phosphorylation was equivalent between both 2D and 3D culture conditions, both Src and FAK displayed reduced phosphorylation in 3D cultures (Figure 5A and B). However, global activation of these molecules was not affected by the presence of NEDD9 as there was no difference in Src and FAK phosphorylation between WT MEF and NEDD9−/− cells in either 2D or 3D cultures. In contrast, there is a striking differential effect on MLCK activity. Under 2D culture conditions, MLC-phosphorylation appears to be significantly reduced in NEDD−/− MEFs. Conversely MLC-phosphorylation is significantly increased in NEDD9−/− MEFs grown in 3D collagen gels (Figure 5A and B). The observed increased MLC phosphorylation in the NEDD9−/− MEFs in 3D collagen gels agrees with earlier reports showing that NEDD9 depletion is accompanied by activation of MLCK [9]. Following the differential signalling effects in 2D versus 3D, we questioned whether NEDD9 is also differentially regulated in 2D versus 3D. NEDD9 is detected as two distinct phospho-forms of 115 kDa and 105 kDa apparent molecular mass [36]. We have previously shown that 115 kDa NEDD9 is formed in response to integrin receptor stimulation [11]. Analysis of the NEDD9 phospho-forms in WT MEFs revealed that 115 kDa NEDD9 is significantly decreased in 3D cell cultures (Figure 5A and B).

**Figure 6:**
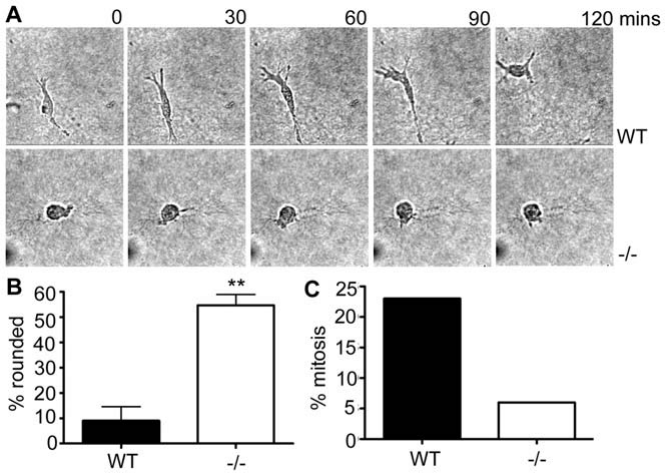
NEDD9 −/− MEFs adopt a rounded morphology. A. Time-lapse series of WT and NEDD9−/− MEFs showing cellular morphologies in 3D collagen gels. B. Percentage of cells exhibiting a rounded phenotype. ** *p*<0.001, Students' *t*-test. C. Mitotic events are decreased in NEDD9−/− fibroblasts. The number of cells observed to undergo mitosis in 3D collagen gels during 6 hours of filming were quantified and expressed as a percentage of the total cells analysed. Data shown are the average of two independent experiments.

During observation of the time-lapse imaging series, it was noted that the majority of the NEDD9−/− MEFs had a rounded appearance in 3D collagen gels and quantification of the percentage of rounded cells confirmed this (Figure 6A and 6B). We considered whether the previously described mitotic defects associated with altered NEDD9 expression levels [31], [32] might account for cell rounding. However this was not the case, as we observe fewer mitotic events in NEDD9−/− cells in 3D collagen gels when compared with WT controls (Figure 6C). Based on the increased rounding and increased levels of MLC phosphorylation, we questioned whether NEDD9 depletion stimulated a switch to amoeboid invasion in the MEFs, as has previously been shown in other cell types [9]. However, the reduced speed of NEDD9−/− MEFs suggests that the observed rounding does not reflect a switch to an amoeboid mode of migration. The rounded shape of the NEDD9−/− MEFs, suggested that these cells may have reduced interaction with the surrounding matrix.

### NEDD9 increases adhesion strength and β1 integrin activation

**Figure 7:**
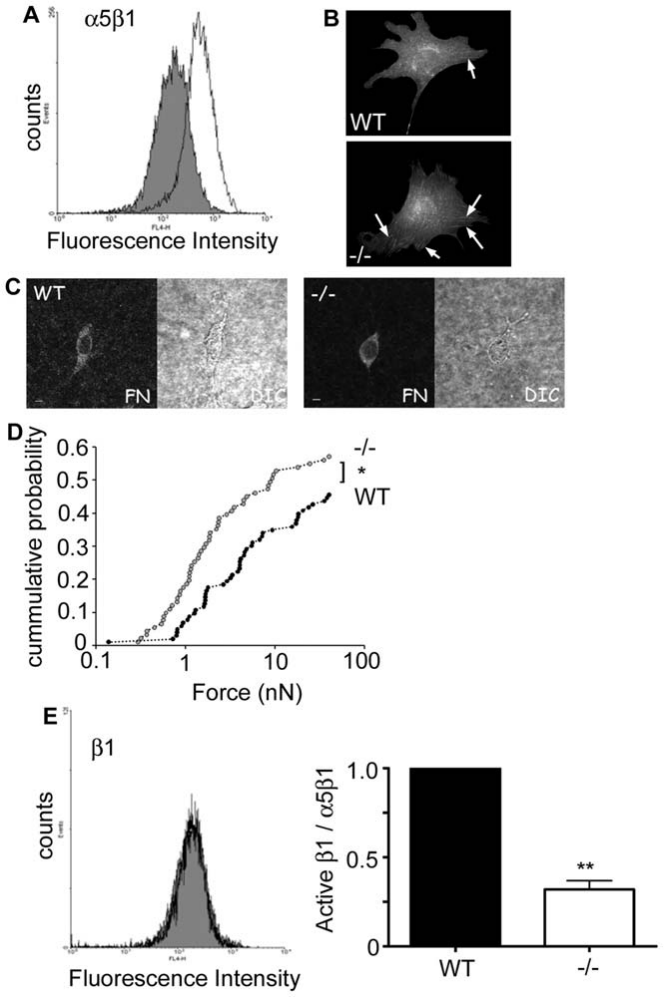
Adhesion strength is decreased in NEDD9−/− MEFs. A. Surface expression of α5β1 determined by FACs analysis in WT (grey) and NEDD9 −/− (white) MEFs. B. α5β1 distribution in cells grown under 2D conditions. Arrows point to α5β1-positive focal adhesions. C. Fibronectin (FN) staining and Differential Interference Contrast (DIC) images of WT and −/− cells in 3D collagen gels. D. Fraction of detached fibronectin-coated beads versus pulling force. Adhesion strength was lower in NEDD9−/− MEFs (*grey circles*, n = 91) compared with WT MEFs (*black circles*, n = 103). E. The level of activated β1 (detected using β1 antibody clone 9EG7) normalized to α5β1 and expressed as fold change relative to the WT control. FACs data are the average ± SEM from 5 experiments repeated on separate days. * *p*<0.05, *** *p*<0.001, Students' *t*-test.

**Figure 8:**
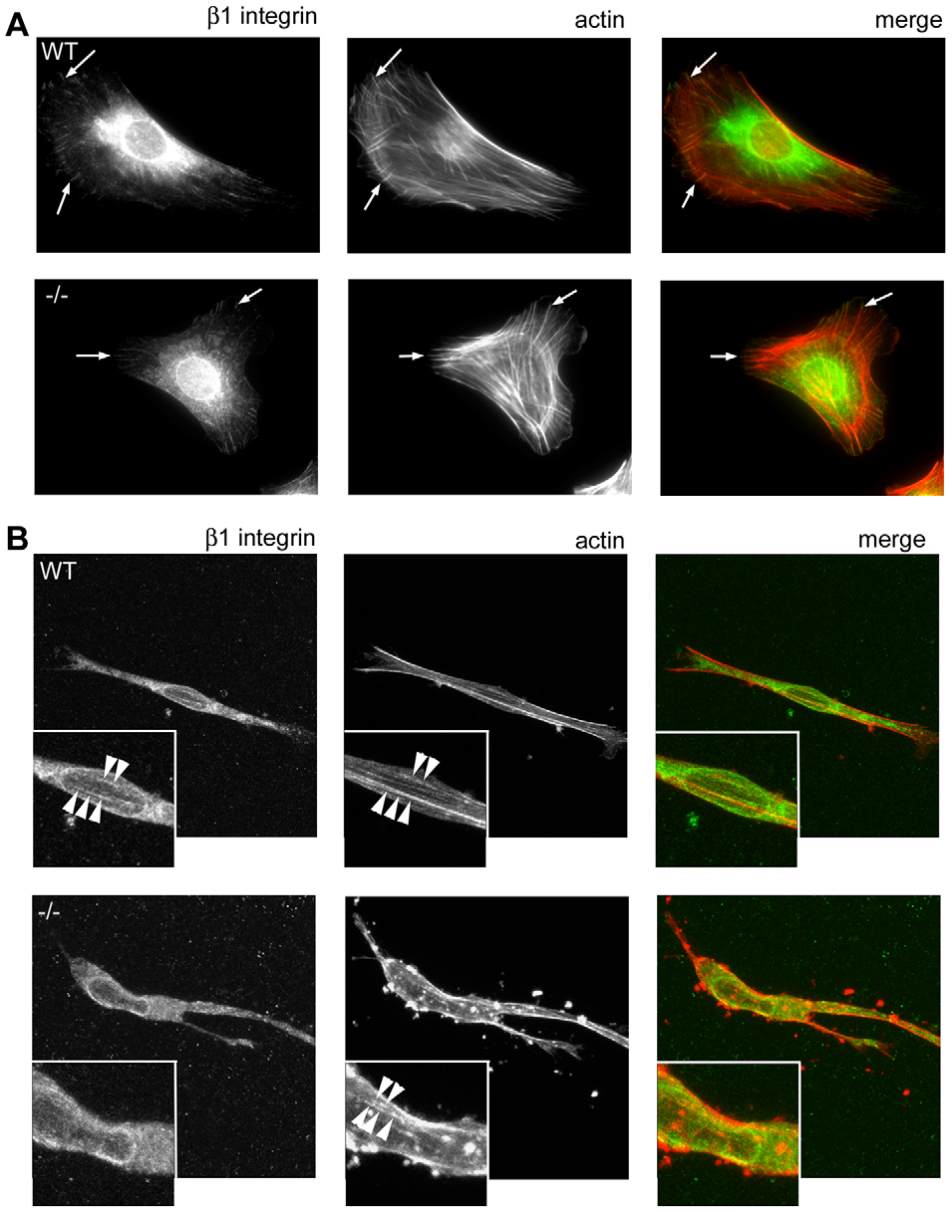
Reduced stress fibres and activated β1 in NEDD9−/− MEFs in 3D. A. Activated β1 integrin (detected using β1 antibody clone 9EG7) and actin (detected with fluorescently-tagged phalloidin) immunostaining of cells grown in 2D conditions. Merged images show colour overlays of β1 (green) and actin (red). Arrows point to examples of active β1 integrin associated with the ends of actin stress fibres. Cells were imaged by wide-field microscopy. B. Activated β1 integrin and actin immunostaining of cells grown in 3D collagen gels. Boxed insets shows magnified regions containing actin stress fibres. Arrow heads point to actin stress fibres. Images represent single confocal z-slices.

Following the demonstration that NEDD9 −/− MEFs have reduced speed and persistence and are more rounded in 3D collagen gels, we questioned whether NEDD9 may regulate the cell adhesion force to the extra-cellular matrix. We first examined the expression of integrin receptors and found little detectable expression of the α2 integrin sub-unit of collagen receptors (data not shown) as has been previously reported for MEFs [21]. By comparison, the cells were strongly positive for the α5β1 fibronectin receptor and indeed there was a striking increase in the expression of α5β1 on the NEDD9 −/− MEFs (Figure 7A). Correspondingly, immunofluorescence analysis of the distribution of α5β1 in fixed cells revealed greater organization of α5β1 in focal adhesions (Figure 7B). Although the 3D cultures are collagen-based, fibronectin is present both in the added serum and can also be secreted by the MEFs. By immunofluorescence we confirmed the presence of fibronectin in the 3D gels (Figure 7C). Therefore, we analysed adhesion force to fibronectin by incubating cells with fibronectin-coated magnetic beads and exposing them to increasing, lateral pulling force in the range of 0 to 40 nN. The force at which a bead detached, if at all, was recorded. Importantly, significantly less force was required to detach the fibronectin-coated beads from the NEDD9−/− cells than from the control cells (Figure 7D). Given that there was reduced adhesion force despite high levels of α5β1 expression, we questioned whether this reflects decreased inside-out activation of β1 integrin receptors. Indeed we observe a striking suppression of activated β1 integrin receptor (clone 9EG7) on the NEDD9−/− MEFs relative to the total levels of α5β1 expression (Figure 7E). Thus, despite elevated α5β1 integrin receptor surface expression, receptor activation was inhibited. We therefore next determined the sub-cellular distribution of activated β1 integrin. As the antibodies to the α5β1 integrin and activated β1 integrin are from the same species it was not possible to co-stain for these two populations. However, in agreement with the surface expression data (Figure 7E), comparison of the staining patterns for these two antibodies reveals that while there is greater organization of α5β1 in focal adhesions in the NEDD9−/− MEFs (Figure 7B), there is no corresponding increase in the organization of activated β1 integrin (Figure 8A). In 3D collagen gels the WT MEFs display robust stress fibre formation and activated β1 integrin co-localizes with stress fibres (Figure 8B). In contrast, the majority of the NEDD9−/−MEF cells were rounded and lacked obvious stress fibres. In those NEDD9−/− MEFs in which stress fibres were evident, there is no corresponding organization of activated β1 integrin (Figure 8B). Thus, together these data suggest that lack of NEDD9 may decrease 3D cell speed by reducing integrin receptor activation and adhesion force to the ECM.

## Discussion

Previous studies have used MEFs derived from mice in which focal adhesion molecules have been genetically deleted to investigate the function of these molecules in focal adhesion dynamics [15]. In the present study, we have used the same approach to investigate the role of NEDD9 and show that the lack of this protein appears to increase focal adhesion turnover in these cells, accompanied by increased cell migration speed in 2D. Thus, in contrast to most other molecules that have been investigated to date, it appears that NEDD9 may suppress focal adhesion disassembly. Further, despite NEDD9 depletion causing increased 2D migration speed, this conversely decreased 3D migration speed.

Our data suggesting that NEDD9 suppresses focal adhesion disassembly is intriguing in light of reports that p130Cas conversely stimulates adhesion turnover [15]. A key difference between these two proteins is the presence of a poly-proline domain in p130Cas C-terminus that is not found in NEDD9. Both proteins contain a consensus Src SH2 binding site in their C-termini, but in p130Cas this site together with the poly-proline domain creates a bipartite binding site for the Src SH2 and SH3 domains respectively [33]. Through the bipartite Src binding motif, p130Cas is thought to amplify Src kinase activity [34], which is a major regulator of focal adhesion disassembly [35]. Additionally, the poly-proline domain of p130Cas has been directly implicated in focal adhesion disassembly [6]. Therefore, the absence of the poly-proline motif in NEDD9 may be one explanation for the differences of these two highly similar proteins on focal adhesion stability in MEFs. In the present study we show that while there is no change in the total levels of Src or FAK phosphorylation in the absence of NEDD9, phosphorylation of the Src/FAK substrate paxillin is increased at focal adhesions, suggesting small increases in localized Src or FAK activity specifically at focal adhesion in these cells. However, reports that Src and FAK activity are reduced in mouse mammary tumours derived from a NEDD9−/− mouse [26] suggest that NEDD9 regulation of these kinases is complex and may depend on cell-type and tumorigenic status.

Similar to our results for NEDD9−/− MEFs, NEDD9 depletion from MCF10a breast epithelial cells and granulocytes also led to increased 2D cell migration [22], [23]. The granulocyte data is particularly interesting, given that lymphocytes isolated from the same NEDD−/− mice conversely display decreased 2D cell migration [23], in keeping with studies in a variety of cancer cell types showing that NEDD9 depletion inhibits migration in scratch wound healing assays [24]–[27] and transwell migration assays [28], [29]. It was suggested that the differential effects of NEDD9 knock-out on granulocyte versus lymphocyte populations may be due to differing expression of the two discrete NEDD9 phospho-forms [23]. Related to this, we have recently established that the NEDD9 phospho-forms have discrete functions in cell spreading [36], [37]. In the present study we demonstrate reduced levels of 115 kDa NEDD9 in cells grown in 3D collagen gels therefore reflecting differential control of NEDD9 phosphorylation in 2D versus 3D conditions. Thus NEDD9 phospho-form expression may play a role in determining the function of NEDD9 as a regulator of focal adhesion turnover. Alternatively, the biphasic response between cell adhesion and migration in 2D [38] means that the consequence of altered focal adhesion turnover rates for final cell speed will depend on whether cells initially had optimal adhesion for the fastest rate of migration. As focal adhesion dynamics are “tuneable” the expression levels of an adhesion stabilizer such as NEDD9 may therefore give different speed readouts in a 2D environment.

In contrast to the results seen in 2D migration assays, we find that NEDD9 depletion significantly reduced migration speeds of MEFs in 3D collagen gels. The critical role of integrin-mediated interaction with the ECM in 3D migration is indicated by studies showing decreased β1 integrin receptor clustering accompanying the switch to amoeboid migration [39]. In the present study we show that cells with genetically depleted NEDD9 have reduced cell adhesion force to fibronectin and the inside-out activation of β1 integrin receptors. These data extend previous findings that NEDD9 regulates Rac-mediated induction of mesenchymal migration and invasion [9], by suggesting that NEDD9 may also determine interaction with the ECM. Further, our data have confirmed earlier reports that MLCK activity is increased following NEDD9 depletion in 3D collagen gels, but in addition, we now show that MLCK activity is conversely decreased under 2D culture conditions. Notably, the data reported here for NEDD9 are highly similar to those previously reported for vinculin. Vinculin has also been suggested to be a negative regulator of adhesion turnover [19] and vinculin knockout fibroblasts have increased 2D migration speed, but decreased 3D migration and invasion [21]. It has previously been suggested that vinculin activation leads to an open conformation with tight affinity for talin, thus reducing focal adhesion turnover [20]. Subsequent connections to the actin cytoskeleton allow the transmission of acto-myosin forces through the adhesion site which results in integrin activation. There was little evidence of activated β1 integrin co-localising with stress fibres in the NEDD9−/− MEFs. Thus a picture is emerging to suggest that molecules which regulate cell adhesion force to the ECM may determine the traction force required for cells to negotiate the surrounding 3D ECM. An important question this raises is whether NEDD9, similar to the function of vinculin [40], impacts the formation and contractility of actin stress fibres and/or, whether NEDD9 mechanically couples contractile forces to the focal adhesions.

The role that adhesion proteins such as NEDD9 play in the regulation of cell migration in 3D contexts is still only poorly understood. Our results suggest that NEDD9 may stabilize focal adhesions and increase adhesion force to the extra-cellular matrix, providing new insight into the function of NEDD9 in 3D cell migration. NEDD9 expression is highly restricted [12] and the protein half-life is short and tightly regulated [36]. As migrating cells negotiate different environments *in vivo* the regulated expression of molecules such as NEDD9 may facilitate the necessary adhesive force required to traverse tissue barriers.

## Materials and Methods

### Cell culture and antibodies

Mouse embryo fibroblasts were maintained in Dulbecco's Modified Eagles Medium (DMEM) (Invitrogen) with 15% Foetal Bovine Serum (FBS) and supplemented with antibiotics (pen/strep, Invitrogen). Immortalized mouse embryo fibroblasts (MEFS) were kindly supplied by Prof Erica Golemis (Fox Chase Cancer Center, Philadelphia USA). Briefly, wild-type and NEDD9 knockout mouse embryos were generated in the Golemis lab through cross-breeding of heterozygous and homozygous null NEDD9 B57/BL6 mice [41]. Embryos were genotyped and wild-type and homozygous knockout MEFs were immortalized with the large T antigen of the SV40 virus. All experiments involving mice were preapproved by the Fox Chase Cancer Center Institutional Animal Care and Use Committee (protocol 06-1, PI Golemis). Growth of cells in 3D collagen gels, media for live imaging and invasion assays through 3D collagen gels have been previously described [42]. The following antibodies were used: anti-NEDD9 (ImmuQuest; clone 2G9 and Cell Signalling Technology; Clone 2G9), anti-FAK (Cell Signalling Technology), anti-phospho-FAK Tyr397 (Life Technologies), anti-Src (Millipore, clone GD11), anti- phosphor-Src Tyr418 (Life Technologies), anti-Myosin Light Chain 2 (Cell Signalling Technology), anti-phospho-Myosin Light Chain 2 Ser19 (Cell Signalling Technology), anti-p42/44 MAPK (Cell Signalling Technology), anti-phospho-p42/44 MAPK Thr202/Tyr204 (Cell Signalling Technology), rat anti-mouse β1 integrin mAb to detect activated β1 integrin (BD bioscience; clone 9EG7), rat anti-mouse α5β1 integrin mAb (Millipore; clone MAB2575), rat IgG isotype control (BD bioscience) and anti-fibronectin (Sigma-Aldrich); HRP-conjugated anti-mouse and anti-rabbit (Amersham and Biorad) and anti-rat Alexa-Fluor 647 conjugate (Molecular Probes, Invitrogen).

### Protein extraction, siRNA and immunoblotting

Conditions of protein extraction from 2D cultures and immunoblotting were carried out as previously described [43]. To extract proteins from cells cultured in 3D collagen gels, gels were first treated with Type I Collagenase (Life Technologies) for 30 min at 37°C and centrifuged at 1000 rpm for 5 min at RT. The cell pellet was washed with 1× PBS then resuspended in lysis buffer and incubated on ice for 30 min. Lysates were centrifuged for 12 min at 13000 rpm at 4°C and the supernatant retained for analysis. Protein concentrations were determined using the BCA Protein Assay Kit (Pierce Biotechnology IL, USA) and protein concentrations equalized prior to loading on gels. Control siRNA and custom-designed NEDD9 siRNA (targeting the sequence CCA GGA CAU UCG CAA CAA A) were both purchased from Dharmacon. 10 nM siRNA was transiently transfected twice at 24 h intervals, using Lipofectamine2000 (Life Technologies) as per manufacturer's instructions.

### Live cell imaging, migration and rounding analysis

Time-lapse images were captured using an ORCA ERG cooled CCD camera (Hamamatsu, SDR Clinical Technology NSW, Australia) and an Olympus IX81 inverted microscope equipped with an environmental chamber heated to 37°C. Transmitted light images were captured at indicated time intervals (40× objective). Cells undergoing division or apoptosis were excluded from analyses. Nuclear translocation was tracked in time-lapse image stacks using Metamorph V6.3 software (Molecular Devices). Random migration analyses were performed on sparsely plated cultures. To quantify cell migration behaviour, cell speeds and directional persistence were determined from the Mean Squared Displacement (MSD) of cells versus time as previously described [40]. Only elongated cells were tracked in the analysis of glioblastoma cell line 3D migration, to exclude apoptotic or mitotic cell populations. Cell morphologies in fixed 3D collagen gels were scored as either elongated (displaying extending protrusions and an elongated cell body) or rounded (displaying a rounded cell body and absence of protrusions). Cells were counterstained with propidium iodide and apoptotic cells excluded from morphological analyses.

### Immunofluorescence and FACs analysis

Paxillin immunostaining of focal adhesions and focal adhesion morphometry analysis was performed as previously described [44]. Cells in 3D collagen gels were fixed, quenched, permeabilised and blocked overnight in 1% fatty-acid free BSA/1% donkey serum/PBS. Gels were then incubated with primary antibodies in PBS/1% donkey serum, washed, followed by addition of fluorescently labelled secondary antibodies and TRITC-Phalloidin and after a final wash cells were imaged under (Olympus FV1000 Confocal Laser Scanning Microscope). FACs analysis of surface integrin expression was achieved following trypsinization to detach cells. Cells were immediately washed (0.5% BSA/0.1% NaN_3_/PBS), incubated with primary antibodies, washed, incubated with fluorescently labelled secondary antibodies and after a final wash stained cells were analysed using a FACSCalibur (Becton Dickinson). The isotype control corrected geometric mean fluorescence of at least 3 separate experiments was compared.

### Magnetic tweezers

1 mg of carboxylated superparamagnetic beads (Microparticles, Berlin) with 5±0.21 µm diameter were coated with 50 µg/ml human fibronectin (Roche, Basel, Switzerland) in 1 ml of carbonate buffer (pH 9.4) for overnight or longer at 4°C. After coating, the beads were washed and stored in PBS. 10,000 cells were plated in 35 mm dishes for 24 hours, and fibronectin-coated beads were then added to the cells and incubated for 30 min at 37°C and 5% CO2. Prior to measurements, the dishes were gently washed with fresh medium to remove unbound beads and then placed on a heated microscope stage. With a magnetic tweezer device, a force that was linearly increasing from 0 to 40 nN over 10 seconds was then applied to the beads. The speed of the force ramp was 4 nN/s. Bright-field images of the cell, the bead, and the needle tip were taken by a CCD camera (ORCA ER Hamamatsu) at a rate of 40 frames per second at 40× magnification. Bead positions were tracked in real time using an intensity-weighted centre-of-mass algorithm. The force value at which a bead detached from the cell surface was recorded.

### Focal adhesion dynamics

YFP-paxillin positive focal adhesions were imaged with an Olympus IX81 inverted microscope, with a 60× (NA 1.35) oil objective, using fluorescence filters of BP 460–495/BP510–550 (GFP) and BP 490–500/BP 515–560 (YFP). Images were captured every 2 minutes for 90 minutes total, 250 ms exposure times. Focal adhesions at the protruding edge of the membrane were analysed by measuring the temporal changes in integrated pixel intensity of an individual focal adhesion. Using MetaMorph V6.1r0 (Molecular Devices, USA), a box was drawn close to the focal adhesion to be analysed (in the cell cytoplasm) and the background corrected by subtracting the average pixel intensity of the boxed region from the entire image as previously described [45]. A polygon was drawn around the focal adhesion at time of maximum intensity and the average pixel intensity of the polygon recorded for all time points. Intensity values (I) were expressed relative to the minimum intensity value (I_o_) for calculating adhesion assembly rates, or were expressed relative to the maximal intensity value (I_max_) for calculating adhesion disassembly rates. Assembly and disassembly rate constants (*k*) were then calculated from the slope of the lines generated by plotting the data as either ln(I/I_o_) or ln(I/I_max_), versus time. Fluorescence ratio imaging of phosphorylated paxillin and semi-quantitative analysis of paxillin phosphorylation at focal adhesions was performed as previously described [4], with the addition of a masking step using Metamorph software, whereby images were first subject to a high-pass filter (sensitivity 1) to emphasize the peripheral cell adhesions. The filtered images were then used as a mask for background subtracted images to high-light the peripheral focal adhesions. Note that the high level of phospho-paxillin antibody reactivity in the nucleus means that the nuclear staining is not filtered out and hence gives a strong signal in the final ratio images.

### Image preparation

Final micrograph images and grey level adjustments were prepared in Adobe Photoshop.

## Supporting Information

Figure S1 **Equivalent expression of fluorescently-tagged fusion proteins at focal adhesions.** Comparison of the peak fluorescence intensity (FI) in arbitrary units (a.u.) for YFP-paxillin positive focal adhesions in either wild-type (WT) or NEDD9 −/− transfected MEFs.
(TIF)
