# Supplementary figures and images for: NEDD9 Stabilizes Focal Adhesions, Increases Binding to the Extra-Cellular Matrix and Differentially Effects 2D versus 3D Cell Migration
Source: PLoS One. 2012 Apr 11;7(4):e35058. doi: 10.1371/journal.pone.0035058 (PMC3324407; doi:10.1371/journal.pone.0035058)

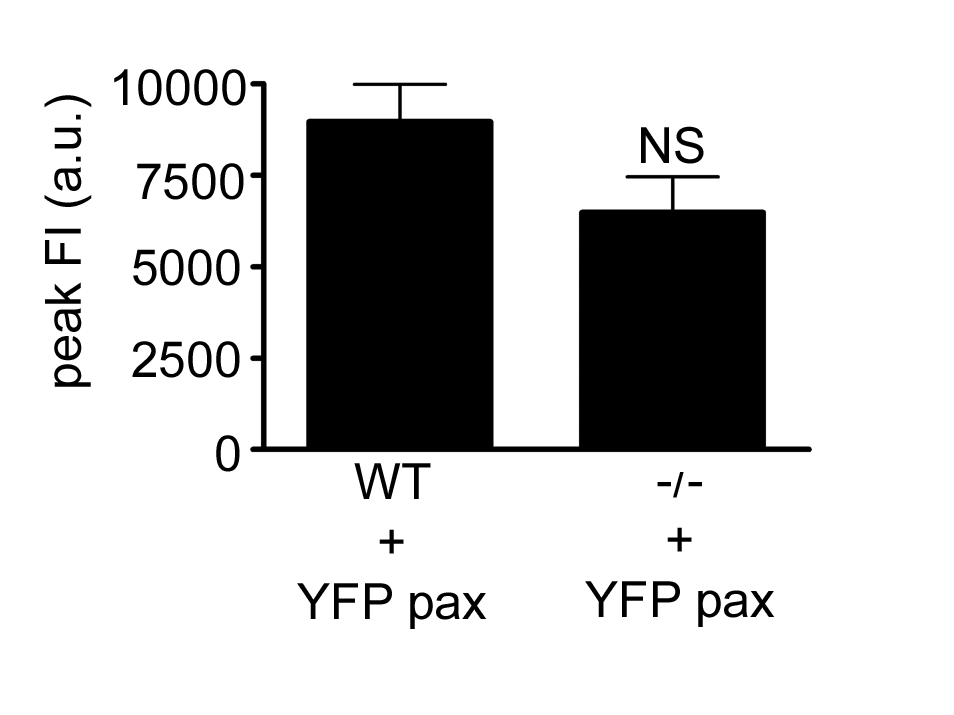

Supplement: Figure S1 — Equivalent expression of fluorescently-tagged fusion proteins at focal adhesions. Comparison of the peak fluorescence intensity (FI) in arbitrary units (a.u.) for YFP-paxillin positive focal adhesions in either wild-type (WT) or NEDD9 −/− transfected MEFs. (TIF) [file pone.0035058.s001.tif]
